# Parkinson’s disease variant detection and disclosure: PD GENEration, a North American study

**DOI:** 10.1093/brain/awae142

**Published:** 2024-07-30

**Authors:** Lola Cook, Jennifer Verbrugge, Tae-Hwi Schwantes-An, Jeanine Schulze, Tatiana Foroud, Anne Hall, Karen S Marder, Ignacio F Mata, Niccolò E Mencacci, Martha A Nance, Michael A Schwarzschild, Tanya Simuni, Susan Bressman, Anne-Marie Wills, Hubert H Fernandez, Irene Litvan, Kelly E Lyons, Holly A Shill, Carlos Singer, Thomas F Tropea, Nora Vanegas Arroyave, Janfreisy Carbonell, Rossy Cruz Vicioso, Linn Katus, Joseph F Quinn, Priscila D Hodges, Yan Meng, Samuel P Strom, Cornelis Blauwendraat, Katja Lohmann, Cynthia Casaceli, Shilpa C Rao, Kamalini Ghosh Galvelis, Anna Naito, James C Beck, Roy N Alcalay

**Affiliations:** Medical and Molecular Genetics, Indiana University School of Medicine, Indianapolis, IN 46202, USA; Medical and Molecular Genetics, Indiana University School of Medicine, Indianapolis, IN 46202, USA; Medical and Molecular Genetics, Indiana University School of Medicine, Indianapolis, IN 46202, USA; Medical and Molecular Genetics, Indiana University School of Medicine, Indianapolis, IN 46202, USA; Medical and Molecular Genetics, Indiana University School of Medicine, Indianapolis, IN 46202, USA; Parkinson’s Foundation, NewYork, NY 10018, USA; Neurology, Columbia University Irving Medical Center, New York, NY 10032, USA; Genomic Medicine, Lerner Research Institute, Cleveland Clinic, Cleveland OH 44106, USA; The Ken & Ruth Davee Department of Neurology, Northwestern University, Chicago, IL 60611, USA; Struthers Parkinson’s Center, Golden Valley, MN 55427, USA; Department of Neurology, Massachusetts General Hospital, Boston, MA 02114, USA; The Ken & Ruth Davee Department of Neurology, Northwestern University, Chicago, IL 60611, USA; Department of Neurology, Icahn School of Medicine at Mount Sinai, New York, NY 10029, USA; Department of Neurology, Massachusetts General Hospital, Boston, MA 02114, USA; Genomic Medicine, Lerner Research Institute, Cleveland Clinic, Cleveland OH 44106, USA; Department of Neurosciences, University of California San Diego, San Diego, CA 92093, USA; Department of Neurology, University of Kansas Medical Center, Kansas City, KS 66160, USA; The Muhammad Ali Parkinson’s Center, Barrow Neurological Institute, Phoenix, AZ 85013, USA; Department of Neurology, Miller School of Medicine, University of Miami, Miami, FL 33136, USA; Department of Neurology, University of Pennsylvania, Philadelphia, PA 19104, USA; Department of Neurology, Baylor College of Medicine, Houston, TX 77030, USA; Centro Cardioneuro Oftalmológico y Trasplante, Santo Domingo 10306, República Dominicana; Medicina Interna, Clínica Unión Médica del Norte, Santiago de los Caballeros 51000, República Dominicana; Neurology, Columbia University Irving Medical Center, New York, NY 10032, USA; Brain Institute, Oregon Health & Sciences University, Portland, OR 97239, USA; Medical and Molecular Genetics, Indiana University School of Medicine, Indianapolis, IN 46202, USA; Fulgent Genetics, Temple City, CA 91780, USA; Illumina Inc., San Diego, CA 92122, USA; Laboratory of Neurogenetics, National Institute on Aging, National Institute of Health, Bethesda, MD 20892, USA; Institute of Neurogenetics, University of Lübeck, 23538 Lübeck, Germany; Clinical Trials Coordination Center, University of Rochester Medical Center, Rochester, NY 14627, USA; Parkinson’s Foundation, NewYork, NY 10018, USA; Parkinson’s Foundation, NewYork, NY 10018, USA; Parkinson’s Foundation, NewYork, NY 10018, USA; Parkinson’s Foundation, NewYork, NY 10018, USA; Parkinson’s Foundation, NewYork, NY 10018, USA; Neurology, Columbia University Irving Medical Center, New York, NY 10032, USA; Movement Disorders Division, Tel Aviv Sourasky Medical Center, Tel Aviv 6423906, Israel

**Keywords:** Parkinson’s disease, genetic testing, genetic counselling, *LRRK2*, *GBA1*, clinical trials

## Abstract

Variants in seven genes (*LRRK2*, *GBA1*, *PRKN*, *SNCA*, *PINK1*, *PARK7* and *VPS35)* have been formally adjudicated as causal contributors to Parkinson’s disease; however, individuals with Parkinson’s disease are often unaware of their genetic status since clinical testing is infrequently offered. As a result, genetic information is not incorporated into clinical care, and variant-targeted precision medicine trials struggle to enrol people with Parkinson’s disease. Understanding the yield of genetic testing using an established gene panel in a large, geographically diverse North American population would help patients, clinicians, clinical researchers, laboratories and insurers better understand the importance of genetics in approaching Parkinson’s disease.

PD GENEration is an ongoing multi-centre, observational study (NCT04057794, NCT04994015) offering genetic testing with results disclosure and genetic counselling to those in the US (including Puerto Rico), Canada and the Dominican Republic, through local clinical sites or remotely through self-enrolment. DNA samples are analysed by next-generation sequencing including deletion/duplication analysis (Fulgent Genetics) with targeted testing of seven major Parkinson’s disease-related genes. Variants classified as pathogenic/likely pathogenic/risk variants are disclosed to all tested participants by either neurologists or genetic counsellors. Demographic and clinical features are collected at baseline visits.

Between September 2019 and June 2023, the study enrolled 10 510 participants across >85 centres, with 8301 having received results. Participants were: 59% male; 86% White, 2% Asian, 4% Black/African American, 9% Hispanic/Latino; mean age 67.4 ± 10.8 years. Reportable genetic variants were observed in 13% of all participants, including 18% of participants with one or more ‘high risk factors’ for a genetic aetiology: early onset (<50 years), high-risk ancestry (Ashkenazi Jewish/Basque/North African Berber), an affected first-degree relative; and, importantly, in 9.1% of people with none of these risk factors. Reportable variants in *GBA1* were identified in 7.7% of all participants; 2.4% in *LRRK2*; 2.1% in *PRKN*; 0.1% in *SNCA*; and 0.2% in *PINK1*, *PARK7* or *VPS35* combined. Variants in more than one of the seven genes were identified in 0.4% of participants.

Approximately 13% of study participants had a reportable genetic variant, with a 9% yield in people with no high-risk factors. This supports the promotion of universal access to genetic testing for Parkinson’s disease, as well as therapeutic trials for *GBA1* and *LRRK2*-related Parkinson’s disease.

## Teaser

Cook *et al.* present results from an ongoing multicentre observational study offering genetic testing with results disclosure and genetic counselling to individuals with Parkinson's disease across North America. Among the approximately 8000 participants who have received results to date, 13% have a reportable genetic variant.

## Introduction

Parkinson’s disease is a neurodegenerative disease characterized by progressive motor disability and non-motor symptoms.^1^ To date, seven genes (*LRRK2*, *GBA1*, *PRKN*, *SNCA*, *PINK1*, *PARK7*, *VPS35*) have been curated by the ClinGen Parkinson’s Disease Gene Curation Expert Panel (PD GCEP) as having a causal relationship with Parkinson’s disease (https://search.clinicalgenome.org/kb/affiliate/10079). Prior studies have identified pathogenic variants in Parkinson’s disease-linked genes in about 5%–10% of people with Parkinson’s disease in the US and Europe.^2-10^ However, only a small fraction of people with Parkinson’s disease receive genetic testing due to lack of awareness among clinicians and patients, cost issues and lack of clinician confidence in providing results to patients.^5,11^ Previous studies have shown a greater likelihood of positive (abnormal) gene test results among those with an earlier age at onset (AAO), positive family history or certain ancestry such as Ashkenazi Jewish, Spanish Basque or North African Berber.^12^ However, recent research indicates that different gene variants may be found in individuals of diverse ancestries from various countries and global regions.^13^

There are several clinical reasons why individuals with Parkinson’s disease might wish to have genetic testing: to aid in diagnosis, to answer the question, ‘Why did I get Parkinson’s disease?’, to inform prognosis, to assist in treatment and life decisions and to clarify the Parkinson’s disease risk for other family members. In addition, there is growing interest within the pharmaceutical industry in genetically targeted precision medicine. Accordingly, in the research and direct-to-consumer (DTC) spaces, there has been a notable increase in genetic testing for Parkinson’s disease over the past decade.^14,15^ Over 10 000 people with Parkinson’s disease have ordered DTC testing from 23andMe that includes health risk assessment for Parkinson’s disease via limited, targeted testing of a single variant, each, within the genes *LRRK2* and *GBA1*.^16^ Genetic information about Parkinson’s disease is being used to determine clinical trial eligibility for gene-specific trials.^1,17^

The genetics of Parkinson’s disease is complex. Counselling issues such as dominant and recessive inheritance, reduced penetrance, phase determination for carriers of two variants for recessive disorders, the uncertainty of the relationship between heterozygous forms of Parkinson’s disease thought to be recessively inherited and disease risk, the risk for both Gaucher disease and Parkinson’s disease in relation to some but not all *GBA1* variants and the possibility of carrying disease-related variants in more than one Parkinson’s disease gene require thoughtful discussion with a clinician skilled in explaining genetic concepts (genetic counsellor or trained neurologist). We have shown that neurologists are uncomfortable with their knowledge of Parkinson’s disease genetics^5^ and access to genetic counsellors in North America is limited.^18^

With this background, the Parkinson’s Foundation, a non-profit Parkinson’s disease research and advocacy organization, launched PD GENEration (PD GENE) as a clinical study in 2019 with the goal of educating and empowering people with Parkinson’s disease by offering Clinical Laboratory Improvement Amendments (CLIA)-certified genetic testing with return of results in the context of genetic counselling at no cost to the participants.^18^ PD GENE addresses potential barriers to testing by providing genetic counselling in either English or Spanish, in local or remote settings. The study has had three phases thus far. In its pilot phase, we verified feasibility of the study and documented both a strong community interest and satisfaction with the testing process among both participants and providers with no significant adverse psychological sequelae in participants.^18^ Here, we report the genetic testing results along with clinical data from the first three phases of the study through June 2023.

## Materials and methods

### Study design and participants

PD GENE is a multi-centre, observational and registry, clinical study (NCT04057794, NCT04994015) offering CLIA-certified genetic testing and genetic counselling to people with Parkinson’s disease in North America,^18^ with an original enrolment goal of 15 000 by 2025. The study was approved by centralized and site institutional review boards (IRBs), as well as the Scientific Review and Executive Committees of the Parkinson Study Group. All participants signed informed consents. The study has had three phases: a pilot phase (600 tested participants) and a clinical phase (the pilot participants plus an additional 1354 tested participants) in which detailed clinical data were collected, as well as the current registry phase with a simplified protocol (6347 tested participants). We include here data from all phases, described in more detail in the Supplementary material. Participants may enrol through their local study site or, remotely, through designated, national enrolling sites with IRB approval to provide telemedicine genetic testing and counselling on a national level or via the Parkinson’s Foundation website https://www.parkinson.org/pdgeneration. Participants are eligible for the study if they meet the Movement Disorder Society (MDS) Clinical Diagnostic Criteria for probable Parkinson’s disease based on examination, chart review or self-report,^19^ are at least 18 years of age, able to provide informed consent in English or Spanish, complete study activities and willing to undergo genetic testing and be informed of their results. In the registry phase, clinical examinations and chart reviews are not performed. Race and ethnicity are obtained by self-report, using categories developed by the US Census Bureau and collected by the study to assess outreach to underserved communities. Self-reported ancestry is collected to evaluate trends in genetic testing yields related to geographic/genealogic descent. Participants are asked to report if a parent, child and/or sibling had a Parkinson’s disease diagnosis established by a physician or by autopsy. All study materials were created in or translated to Spanish and culturally adapted for the Hispanic/Latino community, involving input from the PD GENE Latino Advisory Committee and professional translators. Sites that have bilingual staff are eligible to recruit Spanish-speaking participants. Further details about the processes and protocol of the study can be found at the Parkinson’s Foundation website (https://parkinson.org/pdgeneration).

### Genetic testing and genetic counselling

Following participant consent and sample collection, testing of the genes *LRRK2*, *GBA1*, *PRKN*, *SNCA*, *PINK1*, *PARK7* and *VPS35* is performed at Fulgent Genetics, a CLIA-certified US laboratory. These genes are recognized as major causes of Parkinson’s disease and are included on most commercial Parkinson’s disease genetics panels.^14^ In addition, the seven genes on this panel were previously adjudicated by an independent panel of Parkinson’s disease experts using a recently developed framework supported by the National Institutes of Health (NIH)-funded Clinical Genome Resource (ClinGen)^20^ that found sufficient evidence to support a gene-disease relationship for all of them.

As previously reported,^21^ next-generation sequencing (NGS) and data analysis are performed on genomic DNA. Genomic DNA isolated from accessioned samples (blood or buccal saliva) is prepared into libraries using a customized hybrid capture enrichment protocol, targeting key coding exons and splicing junctions based on IDT xGen Lockdown probe chemistry (Integrated DNA Technologies, Inc., Coralville, IA, USA). Paired-end sequencing is then performed on DNA libraries on the Illumina platform 2500 HiSeq or NovaSeq 6000, using 300 bp reads [size of genomic fragments are 400–500 bp peak size (range 150–900 bp)]. Following alignment to the human genome reference sequence (assembly GRCh37/hg19), variants are detected in regions with at least 10× coverage. For specimens, 99% and 98% of coding regions and splicing junctions (±20 bp of canonical exon splice donor) of the genes listed are sequenced with coverage of at least 10× and 20×, respectively, or by Sanger sequencing. However, the average coverage is usually >100 from this assay, and we achieve a typical coverage >50× (except for *GBA1* exons 9–11). For germline variants, 20× is typically considered sufficient to call a heterozygous variant with an allele fraction of about 50%. In addition, all the variants with quality scores less than 500 (roughly 40× coverage for a heterozygous variant) in the NGS-based panel sequencing are confirmed by targeted Sanger sequencing. The genes are also evaluated for large deletions and/or duplications, and putative deletions or duplications identified are confirmed by quantitative PCR (qPCR) or multiplex ligation-dependent probe amplification (MLPA) by MRC-Holland (Amsterdam, The Netherlands). The analysis of single exon deletions and duplications is performed on the *PRKN* gene. The NGS misalignment analysis is performed on the *GBA1* gene to avoid pseudogene interference. When a potential variant misalignment is identified, long-range PCR is performed to confirm variants and gene conversion.^22^ To verify homozygosity for variants in the *GBA1* gene, for each exon, the lab utilizes two sets of primers to account for possible allele dropout. This method in combination with NGS data and misalignment analyses, greatly reduces the likelihood of allele dropout that would be categorized incorrectly. When a single pathogenic or likely pathogenic variant is identified in a gene with autosomal recessive inheritance, e.g. *PRKN*, *PINK1* or *PARK7*, 100% of coding sequences of that gene are covered either through NGS or Sanger sequencing technologies. For bioinformatics, the Fulgent Germline v2019.2 pipeline is used for analysis.

Variants are initially curated using automated ranking rules and further interpreted manually using locus-specific databases, literature searches and other molecular biological principles. All variants detected in the reportable region (i.e. coding exons and 20 bp flanking introns) are assessed based on the American College of Medical Genetics and Genomics (ACMG) guideline for sequence variant interpretation.^23^ Variants are classified into five-tier categories: pathogenic (P), likely pathogenic (LP), variants of uncertain significance (VUS), likely benign and benign. *GBA1* variants are classified based on their pathogenicity to cause Gaucher disease, except for the *GBA1* c.1093G>A (p.Glu365Lys)/E365K/E326K variant, which is associated with Parkinson’s disease risk and reduced glucocerebrosidase enzymatic activity but not with Gaucher disease.^24,25^ Details regarding ranking rules used for curation and additional methodology applied to the *GBA1* gene are provided in the Supplementary material, Methods section.

Variants deemed reportable in this study include those classified as P/LP, whereas VUS are not reported to the clinician or the participant. There is some controversy about the relationship between certain *GBA1* variants and the development of Parkinson’s disease. We chose to report the *GBA1* c.1093G>A (p.Glu365Lys)/E365K/E326K variant, which is referred to as a ‘risk variant’ with a low penetrance for Parkinson’s disease (about twice the population risk) and no risk for Gaucher disease,^25^ because at the time the study began, individuals with Parkinson’s disease who carried this variant were eligible to participate in a *GBA1*-Parkinson’s disease therapeutic trial. For this reason, this risk variant was felt to be clinically actionable. However, another *GBA1* risk variant, c.1223C>T (p.Thr408Met)/T408 M/T369M, was not reported back to participants as its level of pathogenicity was controversial at study onset but is included as a ‘disease-relevant result’.^24^ Monoallelic P/LP heterozygous variants of autosomal recessive Parkinson’s disease genes such as *PRKN* were considered as reportable, due to the potential implications for reproductive risks in relatives, despite lack of consensus about causation in Parkinson’s disease.^26^

VUS are not disclosed to participants but are catalogued for research use and shared with a global consortium of Parkinson’s disease geneticists and clinicians to centralize and harmonize discussions of VUS identified across multiple studies (ClinGen Parkinson’s Disease Variant Curation Expert Panel: https://clinicalgenome.org/affiliation/50079/). Participants in PD GENE are asked if they wish to give permission to be recontacted for further research based on their Parkinson’s disease gene status and can opt in to receive additional genetic information from the study, such as VUS that are later classified as P/LP. Curated gene variants will be deposited in the NIH ClinGen and ClinVar repositories. Coded, de-identified DNA samples and raw sequence data are stored and are available to researchers upon request.

All participants receive genetic test results (negative or positive) via an in-person or remote genetic counselling session provided by neurologists or certified genetic counselors, at local or nationally enrolling sites. Test results are interpreted by clinicians for participants in the context of their clinical findings and medical and family histories. All participants receive a copy of their gene test laboratory report and a summary of the genetic counselling session. Those with biallelic *GBA1* P/LP variants are referred to local Gaucher centres for further evaluation and counselling.

### Statistical analysis

All statistical analyses were performed using R 4.2.2. For continuous variables, mean, standard deviation (SD), medians and interquartile range (IQR) are provided. For categorical data, the percentage and counts for each category over total number of available participants are provided. To compare differences in clinical measures of interests that are continuous, such as AAO between groups, we used Mann–Whitney test to reduce the type I error due to non-normality of the outcome variables. For the categorical outcomes, such as sex, we used Fisher’s exact test to reduce the type I error due to sparse cell counts (less than five in any cell) or chi-squared test with two-sided test. All *P*-values are reported for two-sided tests.

To provide the results in Tables 1 and 3, our primary analyses, we conducted a total of 33 discovery tests comparing Parkinson’s disease disease-related items. Using Bonferroni correction for multiple testing, our statistical significance threshold was 0.05/33 or 0.0015.

**Table 1:** Demographic characteristics of participants tested

Variable	Tested (*n* = 8301)	Negative (*n* = 7231)	Positive (*n* = 1070)	*P*-value, positive versus negative
Age at enrolment, years, mean (SD), (IQR)	67.4 (10.8), (61.0–74.3)	67.7 (10.7), (61.7–75.0)	65.2^a^ (11.7), (58.0–73.0)	3.63 × 10^−10^
Reported sex, % male (count/total)	59% (4902/8300)	60% (4339/7230)	53%^a^ (563/1070)	4.94 × 10^−6^
Self-reported race % (count/total)	–	–	–	0.239
White	86% (6978/8159)	85% (6059/7111)	88% (919/1048)	–
Alaska Native/American Indian	<1% (16/8159)	<1% (15/7111)	<1% (1/1048)	–
Asian	2% (195/8159)	2% (175/7111)	2% (20/1048)	–
Native Hawaiian/Pacific Islander	<1% (7/8159)	<1% (6/7111)	<1% (1/1048)	–
Black/African American	4% (350/8159)	4% (312/7111)	4% (38/1048)	–
Multiple	3% (225/8159)	3% (204/7111)	2% (21/1048)	–
Self-reported ethnicity % (count/total)	–	–	–	0.114
Hispanic/Latino	9% (707/7925)	9% (601/6895)	10% (106/1030)	–
AAO of PD, years, mean (SD), (IQR)	61.1 (11.1), (54.0–69.0)	61.5 (10.9), (55.0–69.0)	58.3^a^ (12.3), (51.0–67.0)	3.53 × 10^−15^
PD duration, years, mean (SD), (IQR)	5.7 (5.8), (1.0–8.0)	5.6 (5.7), (1.0–8.0)	6.5^a^ (6.6), (2.0–9.0)	2.12 × 10^−5^
Early onset (AAO of PD < 50 years), % (count/total)	16% (1276/8049)	15% (1040/7004)	23%^a^ (236/1045)	9.79 × 10^−10^
High-risk ancestry^b^, % (count/total)	14% (1130/8301)	12% (860/7231)	25%^a^ (270/1070)	4.13 × 10^−28^
First degree relative with PD, % (counts/total)	20% (1579/7994)	19% (1295/6969)	28%^a^ (284/1025)	4.55 × 10^−11^

AAO = age at onset; IQR = interquartile range; PD = Parkinson’s disease; SD = standard deviation.

^a^Statistically significant after multiple test correction (*P* < 0.0015).

^b^Ashkenazi Jewish, Spanish Basque, North African Berber.

## Results

### Participant demographic characteristics

**Figure 1:**
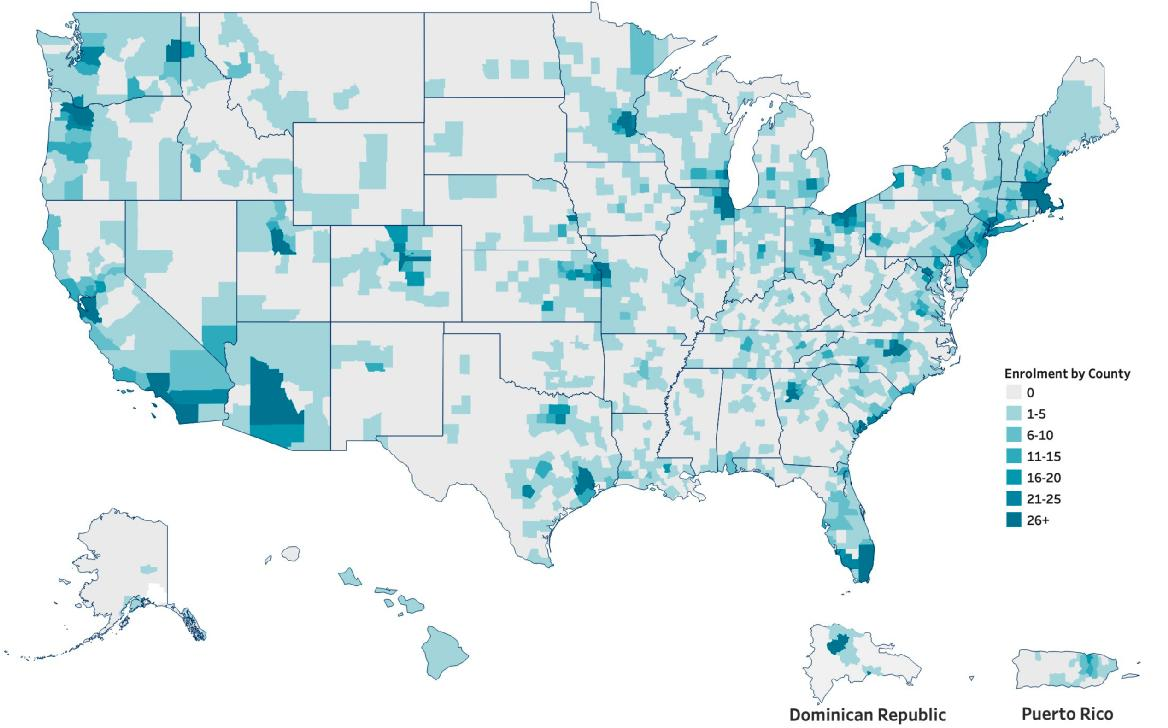
**PD GENEration heat map.** Heat map showing broad enrolment of participants into PD GENEration by county in the US, Puerto Rico and the Dominican Republic. Use of telemedicine allowed for enrolment of participants from all 50 states, representing both urban and rural areas.

More than 10 510 people with Parkinson’s disease across North America have enrolled in the study (Fig. 1), of whom 8301 have completed genetic testing and received results as of 1 June 2023. Population descriptions are detailed in Table 1 and approximate expected frequencies among those who typically participate in research in the US.^27^ We observed that 16% of tested participants had early-onset disease (onset at <age 50). Fourteen per cent were of self-reported high-risk ancestry (Ashkenazi Jewish, North African Berber or Spanish Basque) and 20% reported having a first-degree relative with Parkinson’s disease (Table 1).

### Genetic findings

**Figure 2:**
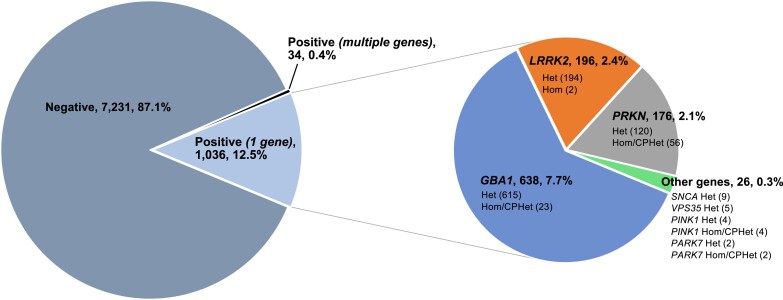
**Reportable results.** Pie chart (*left*) depicting overall positivity rate for reportable variants in a single gene or multiple genes among 8301 participants. *Right*: Categorization of single gene variants for the PD GENEration seven-gene panel. Values are number of participants followed by percentage of total. CPHet = compound heterozygous; Hom = homozygous; Het = heterozygous.

Reportable variants were found in 1070 (12.9%) of the 8301 participants (Table 2). *GBA1* variants were the most frequently identified, followed by *LRRK2* and *PRKN* (7.7%, 2.4% and 2.1%, respectively, and including both single and biallelic variants) (0.4% were multigene variant carriers not included) (Fig. 2). The yield for disease-relevant results, e.g. eliminating those who were heterozygous for *PRKN*, *PINK1* and *PARK7* P/LP variants and adding those individuals who had a c.1223C>T (p.Thr408Met)/T408M//T369M *GBA1* risk variant, was overall 13.4% (1111/8301) and specifically, 10.1% (838/8301) for *GBA1*, 2.4% (196/8301) for *LRRK2* and (56/8301) 0.67% for biallelic *PRKN*.

**Table 2:** Genetic yield by population

Population	Total tested	Reportable variant identified	Yield
All	8301	1070	12.9%
High-risk factors^a^	3453	625	18.1%
No risk factors^b^	4406	399	9.10%
Early onset (AAO of PD<50 years)	1276	236	18.4%
Late onset (AAO of PD ≥50 years)	6773	809	11.9%
First degree relative with PD	1579	284	18.0%
High-risk ancestry^c^	1130	270	23.9%

AAO = age at onset; PD = Parkinson’s disease.

^a^Early-onset PD, high-risk ancestry or first degree relative with PD.

^b^Without early-onset PD, high-risk ancestry and first degree relative with PD.

^c^Ashkenazi Jewish, Spanish Basque, North African Berber.

**Figure 3:**
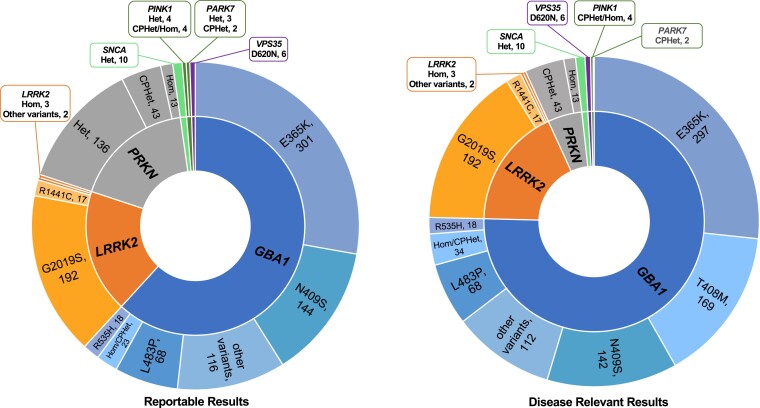
**Variant level results**. Variant level representation of results reportable to participants (*left*), compared to disease relevant results (*right*), which included *GBA1* T408M and excluded heterozygous (Het) carriers of recessive genes. Individuals positive for multiple genes are counted in both gene categories resulting in adjusted totals between graphs. Variant counts are in the heterozygous state unless otherwise specified. Different variant inclusions between graphs resulted in shifts of some individuals from heterozygous to compound heterozygous (CPhet) categories (e.g. two individuals with *GBA1* N409S/T369M represented as N370S on the *left* and compound heterozygous on the *right*). Hom = homozygous.

The most common reportable *GBA1* variants identified were the c.1093G>A (p.Glu365Lys)/E365K/E326K risk variant (3.7%, 307), the c.1226A>G (p.Asn409Ser)/N409S/N370S variant (1.6%, 133) and the c.1448T>C (p.Leu483Pro)/L444P variant (0.8%, 65). Fourteen participants had two variants (phase not determined), including nine with apparent homozygous variants in the *GBA1* gene. Six of these cases involved Gaucher disease-associated variants including c.1226A>G (p.Asn409Ser)/N409S/N370S, representing individuals with likely genotypic Gaucher disease, which was not an exclusion to the study. The most common *LRRK2* variant identified was the c.6055G>A (p.Gly2019Ser) variant (177, 2.1%), including two participants who had homozygous c.6055G>A (p.Gly2019Ser) variants. Heterozygous *PRKN* variants were found in 120 participants: 43 were potentially compound heterozygous (phase not determined) and 13 had homozygous variants. Detected variants in the *PRKN* gene encompassed copy number variants (CNVs) (*n* = 32 unique types) and single nucleotide variants (SNVs)/insertions/deletions (indels) (*n* = 21 unique types). Additional details regarding gene variants observed are described in Figs 2 and 3 and the Supplementary Table 5. Of note, 34 participants (0.4%) had complex results encompassing variants in more than one gene; 32 cases involved the *GBA1* gene, two cases were double heterozygous for the *PRKN* and *LRRK2* genes, and one case had variants in *GBA1*, *LRRK2* and *PRKN* (Supplementary Table 2). VUS data are catalogued and will be reported later.

For those with high-risk ancestry (Ashkenazi Jewish/Basque/North African Berber), early AAO (<50 years) or an affected first-degree relative, the yield for reportable findings was 24%, 18% and 18%, respectively. In those with one or more of these genetic risk factors, 18% received a positive finding (Table 2). When excluding individuals with these predefined genetic risk factors, 9.1% received a positive report.

### Genetic subgroup comparisons

Participant characteristics by genotype are provided in Table 3 and the Supplementary material. Additional subgroup data including data regarding double heterozygotes are provided in Supplementary Tables 2–4. Numbers were too small for some subgroup statistical analyses.

**Table 3:** Participant characteristics by gene variant subgroups

					*P*-values		
Variable	Negative (*n* = 7231)	*GBA1* (*n* = 638)	*LRRK2* (*n* = 196)	*PRKN*, bi (*n* = 56)	*GBA1* versus neg	*LRRK2* versus neg	*PRKN,* bi versus neg
Male: female ratio, (*n* male/*n* female)	1.50 (4339/2890)	1.19 (347/291)	0.81^a^ (88/108)	0.65 (22/34)	0.006	3.19 × 10^−05^	0.002
Self-reported race, count/total (%)					0.617	0.003	0.191
White	85% (6059/7111)	88% (553/628)	91% (172/190)	73% (40/55)	–	–	–
Asian	2% (175/7111)	2% (10/628)	0% (0/190)	9% (5/55)	–	–	–
Black/African American	4% (312/7111)	4% (25/628)	3% (6/190)	5% (3/55)	–	–	–
Multiple	3% (204/7111)	2% (13/628)	2% (3/190	5% (3/55)	–	–	–
Self-reported ethnicity %(count/total)	–	–	–	–	0.260	0.013	6.00 × 10^−5^
Hispanic/Latino	9% (601/6895)	7% (45/612)	14% (27/188)	27%^a^ (15/55)	–	–	–
AAO of PD, years, mean (SD), (IQR)	61.5 (10.8), (55.0–69.0)	58.6^a^ (10.8), (52.0–66.0)	62.9 (9.56), (57.0–69.0)	38.6^a^ (14.8), (27.8–49.0)	2.19 × 10^−11^	0.159	2.37 × 10^−23^
PD duration, years, mean (SD), (IQR)	5.60 (5.70), (1.00–8.00)	5.72 (5.13), (2.00–8.00)	6.04 (5.72), (2.00–8.00)	15.0^a^ (13.2), (4.00–23.0)	0.052	0.173	1.98 × 10^−8^
Early onset (AAO of PD <50 years), %(count/total)	15% (1040/7004)	21%^a^ (130/620)	9% (17/194)	79%^a^ (44/56)	9.41 × 10^−5^	0.018	6.29 × 10^−29^
High-risk ancestry^b^, %(count/total)	12% (860/7231)	19%^a^ (123/638)	62%^a^ (122/196)	4% (2/56)	3.50 × 10^−7^	1.01 × 10^−59^	0.059
First degree relative with PD %(counts/total)	19% (1295/6969)	21% (131/610)	40%^a^ (76/188)	47%^a^ (26/55)	0.084	1.11 × 10^−11^	1.36 × 10^−6^

AAO = age at onset; bi = biallelic (homozygous or compound heterozygous); IQR = interquartile range; PD = Parkinson’s disease; SD = standard deviation.

^a^Statistically significant after multiple test correction (*P* < 0.0015).

^b^Ashkenazi Jewish, Spanish Basque, North African Berber.

#### Self-reported race/ethnicity

Between individuals with positive and negative results, we did not see differences across self-reported race and ethnicity. However, individuals who were Hispanic/Latino were 3.6 times more likely to carry biallelic *PRKN* variants (*P* = 2.07 × 10^−4^) compared with non-Hispanic/non-Latino individuals, among those who tested positive.

#### Age at onset and Parkinson’s disease duration

Those with *GBA1* variants or presumed compound heterozygous or homozygous *PRKN* variants had earlier AAO compared to those with negative results (58.6 and 38.6 versus 61.5 years; *P* = 2.19 × 10^−11^ and 2.37 × 10^−23^), whereas those with *LRRK2* variants did not (Table 3). Carriers of *SNCA* variants had an earlier AAO compared with those with negative results (49.2 versus 61.5 years), though the numbers were too small for statistical comparison. The Parkinson’s disease duration was significantly greater among those with presumed compound heterozygous or homozygous *PRKN* variants.

#### Male to female ratio

The male to female ratio among those with negative results was 1.50, while both the *LRRK2* and the biallelic *PRKN* groups had more females than males. Significance was reached in the *LRRK2* group compared to non-carriers after correction for multiple comparisons (ratio = 0.81, *P* = 3.19 × 10^−5^) (Table 3).

#### High-risk ancestry and positive family history

There was a greater number of participants with high-risk ancestry among the *LRRK2* and *GBA1* subgroups compared with the negative group (62%, 19% versus 12%; *P* = 1.01 × 10^−59^ and 3.50 × 10^−7^). Those in the *LRRK2* and *PRKN* compound heterozygous/homozygous groups were more likely to have a first-degree relative with Parkinson’s disease (40%, 47% versus 19%; *P* = 1.11 × 10^−11^ and 1.36 × 10^−6^). This was not observed for *GBA1* variants (Table 3).

#### MDS-Unified Parkinson’s Disease Rating Scale, Hoehn and Yahr and Montreal Cognitive Assessment

The results of the clinical [MDS Unified Parkinson’s Disease Rating Scale (MDS-UPDRS), Hoehn and Yahr scale] and cognitive assessments [Montreal Cognitive Assessment (MoCA)] and genetic subgroups are reported in the 1954 individuals from the clinical phase who had available results (Table 4). There were no significant differences between scores of those with negative results versus those in the genetic subgroups (all *P*-values > 0.001). Individuals with *SNCA* variants had lower MoCA scores (mean 22.3), although numbers in this subgroup (*n* = 7) were too small for meaningful analysis.

**Table 4:** Clinical measures and demographics of clinical cohort (*n* = 1954)

Variable	Total (*n* = 1954)	Negative (*n* = 1665)	*GBA1* (*n* = 169)	*LRRK2* (*n* = 51)	*PRKN*, het (*n* = 27)	*PRKN,* bi (*n* = 18)	*SNCA* (*n* = 7)^a^
**Clinical measurement, mean (SD), (IQR)**							
MoCA	26.5 (3.00), (25.0–29.0)	26.5 (2.90), (25.0–29.0)	26.1 (3.40), (25.0–28.0)	27.4 (2.30), (27.0–29.0)	26.2 (2.90), (25.0–28.0)	26.8 (2.20), (26.0–28.0)	22.3 (2.50), (20.5–24.0)
MDS-UPDRS	48.3 (23.1), (31.0–62.0)	47.7 (22.3), (31.0–61.0)	52.5 (25.8), (34.2–66.0)	40.6 (25.6), (28.2–42.0)	51.2 (26.2), (32.0–59.2)	48.6 (33.3), (27.0–48.0)	81.2 (19.0), (68.0–95.0)
Hoehn and Yahr	2.0 (0.7), (2.0–2.0)	2.0 (0.7), (2.0–2.0)	2.0 (0.6), (2.0–2.0)	2.0 (0.7), (2.0–2.0)	1.9 (0.7), (2.0–2.0)	2.3 (1.0), (2.0–2.5)	2.3 (0.5), (2.0–2.5)
**Demographic, mean (SD), (IQR)**							
Age, years	64.7 (10.0), (59.0–72.0)	65.1 (9.80), (59.0–72.0)	62.4^b^(9.70), (56.0–69.0)	66.2 (8.00), (61.0–71.0)	64.8 (9.90), (60.0–71.0)	52.3^b^ (15.2), (37.5–62.5)	54.4 (15.6), (43.0–60.0)
Sex, % males (*n*/total)	57% (1110/1954)	58% (960/1665)	51% (87/169)	47% (24/51)^b^	63% (17/27)	33% (6/18)	57% (4/7)
AAO of PD, years	59.3 (10.7), (53.0–67.0)	59.8 (10.5), (53.0–67.0)	57.2^b^ (10.2), (50.0–65.0)	60.9 (8.0), (55.0–66.5)	57.9 (10.7), (50.0–65.0)	41.2^b^ (15.0), (30.8–49.5)	50.0 (16.7), (39.0–56.5)
Disease duration	5.40 (5.20), (2.00–8.00)	5.30 (5.10), (2.00–8.00)	5.20 (4.80), (2.00–8.00)	5.30 (4.70), (1.50–8.00)	7.00 (6.90), (1.00–11.5)	11.2 (12.2), (2.20–15.0)	4.40 (2.90), (2.00–5.50)

AAO = age at onset; bi = biallelic (i.e. homozygous or compound heterozygous); het = heterozygous (i.e. single variant detected); IQR = interquartile range; MDS-UPDRS = Movement Disorder Society-Unified Parkinson’s Disease Rating Scale; MoCA = Montreal Cognitive Assessment; PD = Parkinson’s disease; SD = standard deviation.

^a^Insufficient count for statistical comparison.

^b^ *P* < 0.001.

## Discussion

In this study we counselled and tested 8301 participants for seven established Parkinson’s disease-related genes, focusing initially on the feasibility and safety of counselling/return of results through the local neurologist versus centralized genetic counselors (pilot phase),^18^ then the expansion of the study to multiple clinical sites in the US (clinical phase; Table 4), followed by a simplified study protocol to permit further geographic, racial and ethnic diversity (registry phase; Tables 1–3 andSupplementary Table 1). In this largest and most geographically diverse North American cohort ever tested, we demonstrate an overall yield of reportable genetic variants in 13% of unselected enrolled participants.

There are only a few large-scale, multinational studies with similar attributes to ours as regards ancestry, recruitment, genotyping of multiple genes including *GBA1* and data analyses, and their results are consistent with our observations, with approximately an overall 14% disease-relevant yield, even though larger gene panels were used.^6,7^ Like our study, variants were most often found in *GBA1* and *LRRK2* at similar rates, approaching 10% and 3%, respectively. The most common variant observed was the *GBA1* c.1093G>A (p.Glu365Lys) variant, also known as the E365K/E326K risk allele.^6,7^ Sequencing data from the UK, as part of the Tracking Parkinson’s study, showed lower yields for pathogenic variants for certain genes such as *LRRK2* (0.9%), whereas the yield for *GBA1* variants was similar.^8,9^ The c.6055G>A (p.Gly2019Ser) variant was the predominant abnormal finding in *LRRK2*, similar to reports of Parkinson’s disease genetic testing results in other European-based cohorts.^6,8^ As expected from published literature,^3,6,8,28^ Parkinson’s disease gene variants were found more frequently in those with earlier AAO < 50 (18%, 236/1276), with a first-degree family history of Parkinson’s disease (18%, 284/1579) or high-risk ancestries (24%, 270/1130) compared with our total population rate (Table 2).

Specifically, variants in *GBA1* and *PRKN* (biallelic) were associated with earlier AAO, and those with *LRRK2* variants were more likely to be self-reported as female, confirming prior observations.^6,12^ Female predominance among *LRRK2* carriers with Parkinson’s disease is not yet fully explained, and our study does not shed further light on this question. Individuals with either *LRRK2* or biallelic *PRKN* variants were more likely to report a first-degree relative with Parkinson’s disease, likely attributed to higher penetrance of these gene variants. Although self-reported race did not emerge as a significant variable, individuals who were Hispanic/Latino were more likely to have *PRKN* variants (biallelic) consistent with observations in the CORE-PD study in which those who were Hispanic were more likely to have *PRKN* variants than non-Hispanic individuals.^12^ Since we did not report back VUS by design, as additional variants are adjudicated in ClinGen/ClinVar using patient materials from this and other large studies, we anticipate that some VUS will be elevated to the status of P/LP and then the positivity rate in our cohort will increase.

The absolute number of people with Parkinson’s disease who tested positive for *PINK1*, *PARK7*, *VPS35* and *SNCA* was extremely low, precluding any detailed comments about genotype-phenotypes or participant characteristics for these groups. In addition, results regarding clinical measures by genetic subgroups should be interpreted with caution due to various limitations in study design. They include the cross-sectional nature of the study, a potential referral bias, presentation in earlier phases of disease, examinations by telemedicine for some participants and informed consent requirements for participants to have the cognitive capacity to provide consent, which had the practical effect of excluding individuals with dementia or a low MoCA score.

We highlight that although we observed a yield of 18% for reportable results in participants with one of three identified genetic risk factors (early AAO, affected first degree relative, high-risk ancestry), notably, the positivity rate was 9% among participants in our cohort with none of these risk factors. As the study expands with plans to query more Parkinson’s disease-related genes, yields in both groups will even be greater. Our results provide compelling data to suggest that genetic testing should not be restricted to high-risk individuals, but rather should be offered to all people with Parkinson’s disease.^3,6,7^ For example, while as expected, *GBA1* and *LRRK2* variants were more common among people of Ashkenazi Jewish ancestry compared with those without any high-risk ancestries, most who possessed these variants were non-Ashkenazi Jewish (Supplementary Table 4). However, where resources are limited, testing can be prioritized according to perceived impact and patient interest. Specifically, in cases of limited resources, we propose prioritizing testing for those who would be interested in acting on positive results, e.g. for clinical trial participation or life/family planning.

Motivations to return genetic results to people with Parkinson’s disease include: (i) people are interested in knowing their genetic status^11,18^; (ii) different genetic forms of Parkinson’s disease can have strikingly different prognoses^4^; (iii) emerging evidence that genotype may be relevant for aspects of clinical care^28^; and (iv) genetic status can determine eligibility for precision medicine clinical trials. To date, there are no US Food and Drug Administration (FDA)-approved interventions to modify disease progression in Parkinson’s disease, so approved treatments are all symptomatic. A potential explanation for the failure of multiple clinical trials to demonstrate disease-modifying effect is that Parkinson’s disease pathogenesis is heterogenous. There is reason to hope that therapies targeting Parkinson’s disease with specific associated genetic variants, such as *GBA1* or *LRRK2* variants, might demonstrate disease modification in a subset of people with Parkinson’s disease.^17^ Our findings show that there are many previously unidentified people with Parkinson’s disease who could qualify for precision medicine trials, especially ones focused on *GBA1* (approaching 10% of all participants) and, to a lesser extent, *LRRK2.*

The PD GENE study has attempted to reduce previously identified barriers to genetic testing in Parkinson’s disease, including cost, physician knowledge and comfort with genetics (through training materials and coursework), the perceived low yield of genetic testing and patient awareness.^5^ Strengths of the study include its large and rapidly growing cohort, streamlining of the protocol and use of telehealth strategies to facilitate enrolment from outside the usual geographic (Fig. 1) and cultural reach of academic centres and inclusion of bilingual clinicians and Spanish language materials. The study is also able to contribute patient materials, genetic test results and expertise to several national and global Parkinson’s disease genetics programs, including ClinGen/ClinVar (described above), the Global Parkinson’s Genetics Project (GP2), the Black and African American Connections to Parkinson’s Disease (BLAAC-PD) and Latin American Research consortium on the GEnetics of Parkinson’s disease (LARGE-PD), each targeting Black/African American and Hispanic/Latino Parkinson’s disease populations, respectively.

Despite early efforts, a weakness of this study remains its relative lack of racial and ethnic diversity. Therefore, our results may not be directly applicable to non-European populations. In addition, we acknowledge that in the data analyses stratified by race that these results are less informative as it is increasingly recognized that race is only a social construct based on physical attributes, not accurately representing genetic differences. We recognize that both self-enrolment and physicians who recruit for this study may be biased towards people with early age at onset, positive family history or high-risk ancestry, resulting in some degree of enrichment. However, the study continues to encourage all people with Parkinson’s disease who are interested in testing to participate and to encourage study sites to enrol all-comers, removing these historic barriers to testing. We further acknowledge that the vast majority of study participants received a negative result and that additional genetic research is required to identify risk factors not investigated here (e.g. polygenic risk score). As noted earlier, the interpretation of reported clinical measures by genetic subgroups of the smaller PD GENE cohort were limited by the study design.

In the future, we plan to enhance our ongoing efforts to enrol underrepresented populations and to help improve the understanding of Parkinson’s disease gene variants in these groups. To address this, the PD GENE study has since added the site Morehouse College of Medicine/Grady Hospital in Atlanta, Georgia (traditionally Black/African American) and sites in Puerto Rico and the Dominican Republic. More recently, the study has expanded access to native Hawaiian populations by partnering with Queen’s Medical Center in Honolulu, Hawaii. In addition, PD GENE samples will be included as part of the Aligning Science Across Parkinson’s Global Parkinson’s Genetic Program (ASAP-GP2), in which individuals will be genotyped with the Global Diversity Array (plus Neurobooster)^29^ for almost 2 million variants genome wide. GP2 will use this information to address both global and local ancestry, and this will shed some light on the admixture present in our cohorts, as well as determining ancestral origins of the pathogenic variants identified in PD GENE. This will also allow for a greater ability to characterize unique variants across populations looking for any enrichment.

In summary, this large study of genetic testing for Parkinson’s disease in North America and Caribbean sites confirms a relatively high rate of positive (abnormal) results, approximately 13% for reportable or disease-relevant variants. Positive results were observed in up to 18% in people with genetic risk factors such as early AAO, high-risk ancestry or an affected first degree relative, but also 9% in people lacking any of these risk factors. As trials of gene-specific potentially disease-modifying treatments have begun, and genetic results may impact disease prognosis, possibly for management, and with certainty and clarity related to familial risks, we believe that clinical genetic testing should be offered to all people with Parkinson’s disease to empower them to act upon their genetic findings. Several strategies are underway to enhance the recruitment of other underserved populations into the last third of the study’s planned cohort.

## Supplementary Material

awae142_Supplementary_Data

## Data Availability

Deidentified data will be made available to qualified researchers who submit and provide a valid research question. Enquiries should be directed to R.N.A.

## Appendix 1

PD GENEration Collaborators

Study Core Collaborators

Rebeca De Leon, Amasi Kumeh, Zachary Meyer, Anny Coral-Zambrano, Min-Jae Kim, Camilla Ruiz, Yun Lu, Elisa Ahmanson, Andra Matthews, Sarah Lawrence, Yun Lu, Susan Li, Uma Ragunathan, Deborah Baker, Betty Lyda, Ken Eaton, Olga Pikul, Ett Muneath, Karen Hodgeman, Yan Meng, Rachel Blake, Laura Heathers, Michelle Totten, Amanda Miller, Malia Rumbaugh.

Site Investigators

Rajesh Pahwa, William De Jesus, Josh Shulman, David Simon, Hemant Pandey, Okeanis Eleni Vaou, Anna Hohler, Ihtsham ul Haq, Henry Moore, Stuart Isaacson, Jason Aldred, Ariane Park, Steven Gunzler, Jill Ostrem, Carlie Tanner, Deborah Hall, Rachel Saunders-Pullman, Matthew Barrett, Chantale Branson, Karen Blindauer, Tsao-Wei Liang, Gonzalo Revuelta, Elizabeth Zauber, Julie Schwartzbard, Tritia Yamasaki, Rohit Dhall, Danielle Englert, Giulietta Riboldi, Annie Killoran, Kathleen McKee, Nina Browner, Connie Marras, Peter Lin, Irene Richard, Amy Hellman, Gian Pal, Kelly Mills, John Morgan, Mustafa Siddiqui, Jeanne Feuerstein, Jeffrey Cooney, Joseph Savitt, Tarannum Khan, Houman Homayounh, Stephen Lee, Harini Sarva, David Hinkle, Luis Forastieri, Frances Velez, Angel Vinuela, Thomas Beach, Charles Adler.

Coordinators

Karen Williams, Max Galarce, Lucas George, Rachel Lewandowski, Evalyn Mackenzie, Amanda Chan, Alia Neibaur, Kellie Keith, Emily Leonard, Gita Golonzka, April Langhammer, Sherry Neisen, Hiba Saade, Jamie Fong, Ruby Rendon, MaryBeth Serrano, Mansi Sharma, Arjun Laud, Isabella Montanaro, Tyler Tribble, Lisa Damron, Neda Almassi, Whitney Hartstone, Aine Russell, Hannah Babcock, Jerrod Cook, Giulia Andrews, Sean Guardado, Kaitlyn Ramsey, Savannah Reis, Claudia Cano, Claire Hennum, Amanda Kiefer, Katherine Ambrogi, Victoria Klee, Victoria Miller, Aaron Daley, Marc Rosenbaum, Deborah Raymond, Maya Rawal, Virginia Norris, Tazrin Rahman, Sherline Sauveur, LaShawn Baker, Paula Phabia-Millbrook, Marie Mejaki, Michelle Rochman, Sandra Wilson, Conner Mroz, Lauren Perrey-Moore, Jonathan Castano, Renee Wagner, Michael Nsoesie, Kimberly Gamble, Kelly Astudillo, Heena Olalde, Min-Jae Kim, Geidy Serrano.

Study Partners

Julia Shirvan, Judith Peterschmitt, Pablo Sardi, Cornelis Blauwendraat, Christine Klein, Parkinson Study Group.
