# Supplementary material for: Parkinson’s disease variant detection and disclosure: PD GENEration, a North American study
Source: Brain. 2024 Jul 30;147(8):2668–79. doi: 10.1093/brain/awae142 (PMC11292896; doi:10.1093/brain/awae142)
Supplement: awae142_Supplementary_Data [file awae142_supplementary_data.zip › brain-2023-02602-File009.pdf]

Supplement

Table of Contents **Page**

Methods

Description of PD GENEration Study Phases (pilot, clinical, registry) 2

GBA1 Testing Methodology 2

Variant Calling and Interpretation Resources 2

Table 1. Differences Between the PD GENEration Phases 3

Table 2. Participants with Variants in Multiple Genes 3

Table 3. Clinical Characteristics of Double Heterozygotes 3

Table 4. Frequency and Number of Reportable Variants in *GBA1*/*LRRK2* by Ancestry 3

Table 5. Types and Counts of Reportable/Disease-relevant Variants 4-6

References 7

Methods

Description of PD GENEration Study Phases (Pilot, Clinical, Registry):

The initial phase of the study was a multicenter pilot study aiming to enroll 600 people with Parkinson's disease (PD) for genetic testing and genetic counseling across seven Parkinson Study Group sites in the U.S., with the objective to assess the feasibility of, and interest in, PD genetic testing[1]. Upon completion of the pilot, the clinical phase of the study was launched seeking to enroll 2,000 people with PD across additional U.S. sites, assessing the frequency of reportable variants and gathering clinical phenotype data on genetic subgroups. During the study's pilot and clinical phases, demographics, medical and family histories, and clinical data from neurological assessments (e.g., MoCA [Montreal Cognitive Assessment], MDS-UPDRS [Movement Disorder Society-Unified Parkinson's Disease Rating Scale]) were collected.

GBA1 Testing Methodology

GBA1 and *GBAP1* share 87-100% sequence similarity in their 12 exons. Changes in gene-specific nucleotides in *GBA1* result in several well-established pathogenic variants. In brief, the lab measures the coverage ratio between the target gene *(GBA1)* and the corresponding pseudogene *(GBAP1)* (Gene-to-Pseudogene ratio, G2P). They define the misalignment index (MI) as the natural logarithm of G2P. An ideal G2P ratio is 1, and an ideal MI is 0 (ln 1). If one copy of the pseudogene reads is misaligned to the target gene, the G2P ratio is expected to be 3.0 (3/1), and the expected MI is approximately 1.1 (ln 3). Conversely, if half of the target gene reads are aligned to the pseudogene, the expected G2P ratio is 0.33 (1/3), and the expected MI is approximately -1.1 (ln 0.33). After estimating the gene and pseudogene copy numbers and adjusting the MI thresholds, it can be determined whether an observed MI value is within the normal range and proceed with routine curation. A high MI in a target region of a sample indicates possible false-positive SNVs from the pseudogene, and long-range PCR (LR-PCR) is required to confirm any reportable variants. In contrast, a low MI indicates variants in gene-specific nucleotides (GSN) (the distinct nucleotides between a gene and its pseudogene) and possible false-negative variants. If the variants at GSNs or any observed variant in the pseudogene could be reportable if mapped to the target gene, LR-PCR is recommended for confirmation.

Variant Calling and Interpretation Resources

Our ranking rules take into account external annotation and our internal curation using ACMG guidelines. All variants that potentially have enough evidence for being LP/P based on ACMG guidelines will be given high ranks, prioritized, and manually curated. Curation is based on previous curations (internal and external databases), impact (frameshift, missense, etc.), population frequency, and *in silico* prediction scores (e.g., conservation, splicing). Per the prioritization and curation triage rules, all variants that could reasonably be considered VUS, LP, or P are reviewed by a genomic analyst. Variants with a high likelihood of being classified as B or LB are ranked at the bottom and typically not reviewed. These include variants previously classified as B/LB with high minor allele frequency or has a synonymous consequence without conservation or splice prediction.

Variant Callers: Sentieon Haplotyper 201808.05 and INDELseek (Au CH, Leung AYH, Kwong A, Chan TL and Ma ESK, 2017)

Annotators: Alamut, ClinVar, HGMD

Variant interpretation resources: UCSC, PubMed, NCBI, OMIM, gnomAD, Clingen, GenCC (The Gene Curation Coalition), Decipher, Genetics Home, ClinVar, and DGV. ACMG guideline: Standards and guidelines for the interpretation of sequence variants: a joint consensus recommendation of the American College of Medical Genetics and Genomics and the Association for Molecular Pathology.[2] Genet Med. 2015 May;17(5):405-24.

Please contact author, RNA, to request a list of primers used and/or a bed file documenting the sequences examined.

GBA1 primers and bed files are available through the following link (Password: PDGENE2023).

Table 1. Differences Between PD GENEration Phases – Pilot, Clinical and Registry Phases

Study Phase	Enrollment goals, cumulative	7 gene NGS with del/dup (CLIA/CAP)	Bank DNA, exome data	Post-test genetic counseling	Demographic data collection	MDS-UPDRS	MoCA	Patient, provider surveys
**Pilot**	600	✓	✓	✓	✓	✓	✓	✓
**Clinical**	2000	✓	✓	✓	✓	✓	✓	✓
**Registry**	15000	✓	✓	✓	✓			

CLIA/CAP, Clinical Laboratory Improvement Amendments/ Certified Analytics Professional; MDS-UPDRS, Movement Disorder Society-Sponsored Revision of the Unified Parkinson's Disease Rating Scale; MoCA, Montreal Cognitive Assessment; NGS, next-generation sequencing

Table 2. Participants with Variants in Multiple Genes

Double/triple gene variants observed	Number of participants
GBA1, LRRK2	15
GBA1, PRKN	13
GBA1, SNCA	1
GBA1, VPS35	1
GBA1, PARK7	1
GBA1, LRRK2, PRKN	1
LRRK2, PRKN	2

Table 3. Clinical Characteristics of Double Heterozygotes[a]

Variable	***GBA1*** **N409S and** ***LRRK2*** **G2019S (n=8)**	***GBA1*** **other[b] and** ***LRRK2*** **G2019S (n=5)**
**AAO of PD, mean (SD), (IQR)**	68.8 (13.1), (57.4-78.5)	64.4 (13.0), (53-71)
**PD duration, mean (SD), (IQR)**	5.80 (4.2), (2-9)	0.60 (0.5), (0-1)
**Male: female ratio, (n male/female)**	1.67 (5/3)	0.67 (2/3)
**High-risk ancestry[b], count/total %**	7/8 (88%)	4/5 (80%)
**1st degree relative with PD, count/total %**	3/8 (38%)	4/5 (80%)

[a]Common/historic variant names used
[b]Excludes *GBA1* risk alleles E326K and T408M
[c] High-risk ancestry: Ashkenazi Jewish, Spanish Basque, North African Berber
AAO, age at onset; PD, Parkinson's disease

Table 4. Frequency and Number of Reportable Variants in *GBA1* and *LRRK2* by Ancestry

Ancestry	***GBA1*****, n (%)**	***LRRK2*****, n (%)**
**No high-risk ancestry**	515/7171 (7.2%)	74/7171 (1.0%)
**Ashkenazi Jewish**	118/1050 (11%)	119/1050 (11%)
**North African Berber**	0/13 (0.0%)	1/13 (7.7%)
**Spanish Basque**	5/67 (7.5%)	2/67 (3.0%)

Table 5. Types and Counts of Reportable/Disease-relevant Variants

Gene	Nucleotide change	Protein change	Common Abbreviation, Alternate Nomenclature	Count of variants identified	Classification
GBA1	c.1093G>A	p.Glu365Lys	E365K, E326K	316	Risk Allele
GBA1	c.1123C>T	p.Thr408Met	T408M, T369M	181	Risk Allele
GBA1	c.1226A>G	p.Asn409Ser	N409S, N370S	158	Pathogenic
GBA1	c.1448T>C	p.Leu483Pro	L483P, L444P	77	Pathogenic
GBA1	c.1604G>A	p.Arg535His	R535H, R496H	18	Pathogenic
GBA1	c.1342G>C	p.Asp448His	D448H, D409	14	Pathogenic
GBA1	c.84dup	p.Leu29Alafs*18	84GG	14	Pathogenic
GBA1	c.115+1G>A	p.?	IVS2+1	11	Pathogenic
GBA1	c.1504C>T	p.Arg502Cys	R502C, R463C	11	Pathogenic
GBA1	c.1265_1319del	p.Leu422ProfsTer4	Leu422fs, L335fs	7	Pathogenic
GBA1	c.887G>A	p.Arg296Gln	R296Q, R257Q	7	Pathogenic
GBA1	c.721G>A	p.Gly241Arg	G241R, G202R	5	Pathogenic
GBA1	c.764T>A	p.Phe255Tyr	F255Y, F216Y	5	Pathogenic
GBA1	c.1297G>T	p.Val433Leu	V433L, V394L	4	Pathogenic
GBA1	c.604C>T	p.Arg202*	R202X, R163X	4	Pathogenic
GBA1	c.1246G>A	p.Gly416Ser	G416S, G377S	3	Pathogenic
GBA1	c.475C>T	p.Arg159Trp	R159W, R120W	3	Pathogenic
GBA1	c.680A>G	p.Asn227Ser	N227S, N188S	3	Pathogenic
GBA1	c.914del	p.Pro305Leufs*31	914C del	3	Pathogenic
GBA1	c.946C>T	p.Arg316Cys	R316C, R277C	3	Likely Pathogenic
GBA1	c.1085C>T	p.Thr362Ile	T362I, T323I	2	Likely Pathogenic
GBA1	c.222_224del	p.Thr75del	T75del	2	Pathogenic
GBA1	c.754T>A	p.Phe252Ile	F252I, F213I	2	Pathogenic
GBA1	c.896T>C	p.Ile299Thr	I299T	2	Likely Pathogenic
GBA1	c.928A>G	p.Ser310Gly	S310G	2	Likely Pathogenic
GBA1	c.1052G>C	p.Trp351Ser		1	Likely Pathogenic
GBA1	c.1090G>A	p.Gly364Arg		1	Likely Pathogenic
GBA1	c.1093dup	p.Glu365Glyfs*71		1	Likely Pathogenic
GBA1	c.1192C>T	p.Arg398*		1	Pathogenic
GBA1	c.1240G>T	p.Val414Leu		1	Likely Pathogenic
GBA1	c.1296G>A	p.Trp432*		1	Likely Pathogenic
GBA1	c.1312G>A	p.Asp438Asn		1	Pathogenic
GBA1	c.1312G>T	p.Asp438Tyr		1	Likely Pathogenic
GBA1	c.1353C>A	p.Tyr451*		1	Likely Pathogenic
GBA1	c.166_167dup	p.Cys57Serfs*35		1	Likely Pathogenic
GBA1	c.26_27del	p.Glu9Glyfs*8		1	Likely Pathogenic
GBA1	c.437C>T	p.Ser146Leu		1	Pathogenic
GBA1	c.586A>C	p.Lys196Gln		1	Likely Pathogenic

GBA1	c.589-1G>C	p.?		1	Likely Pathogenic
GBA1	c.635C>G	p.Ser212*		1	Pathogenic
GBA1	c.653G>A	p.Trp218*		1	Pathogenic
GBA1	c.661C>A	p.Pro221Thr		1	Pathogenic
GBA1	c.701G>A	p.Gly234Glu		1	Likely Pathogenic
GBA1	c.703T>C	p.Ser235Pro		1	Pathogenic
GBA1	c.850C>A	p.Pro284Thr		1	Likely Pathogenic
GBA1	c.894C>A	p.Phe298Leu		1	Likely Pathogenic
GBA1	Whole Gene Deletion			1	Pathogenic
LRRK2	c.6055G>A	p.Gly2019Ser	G2019S	198	Pathogenic
LRRK2	c.4321C>T	p.Arg1441Cys	R1441C	17	Pathogenic
LRRK2	c.4309A>C	p.Asn1437His	N1437H	1	Likely Pathogenic
LRRK2	c.4322G>A	p.Arg1441His	R1441H	1	Pathogenic
PRKN	c.823C>T	p.Arg275Trp	R275W	48	Pathogenic
PRKN	Deletion of Exons 3-4			21	Pathogenic
PRKN	Duplication of Exon 2			21	Likely Pathogenic
PRKN	c.155del	p.Asn52Metfs*29	N52fs	16	Pathogenic
PRKN	c.101_102del	p.Gln34Argfs*5	Q34fs	13	Pathogenic
PRKN	c.1289G>A	p.Gly430Asp	G430D	11	Pathogenic
PRKN	c.337_376del	p.Pro113Thrfs*51	P113fs	11	Pathogenic
PRKN	Deletion of Exon 2			11	Pathogenic
PRKN	Deletion of Exon 4			9	Pathogenic
PRKN	Deletion of Exon 3			8	Pathogenic
PRKN	c.719C>T	p.Thr240Met	T240M, T212M, T91M	7	Pathogenic
PRKN	Deletion of Exon 5			5	Likely Pathogenic
PRKN	c.101del	p.Gln34Argfs*10		4	Pathogenic
PRKN	c.1286-3C>G	p.?		4	Likely Pathogenic
PRKN	Deletion of Exons 2-3			3	Pathogenic
PRKN	Deletion of Exons 5-6			4	Pathogenic
PRKN	Deletion of Exon 6			3	Pathogenic
PRKN	Duplication of Exon 3			3	Pathogenic
PRKN	Duplication of Exon 5			3	Likely Pathogenic
PRKN	Duplication of Exons 10-12			3	Likely Pathogenic
PRKN	Duplication of Exons 1-4			3	Likely Pathogenic
PRKN	Duplication of Exons 3-4			3	Likely Pathogenic
PRKN	c.1288G>A	p.Gly430Ser		2	Likely Pathogenic
PRKN	c.1352del	p.Cys451Serfs*190		2	Likely Pathogenic
PRKN	c.167T>A	p.Val56Glu		2	Pathogenic
PRKN	c.633A>T	p.Lys211Asn		2	Likely Pathogenic
PRKN	c.98G>A	p.Arg33Gln		2	Likely Pathogenic
PRKN	Deletion of Exons 2-4			2	Pathogenic
PRKN	Duplication of Exon 7 p.?			2	Pathogenic

PRKN	c.1084-1G>C	p.?		1	Likely Pathogenic
PRKN	c.125G>C	p.Arg42Pro		1	Pathogenic
PRKN	c.1334G>A	p.Trp445*		1	Pathogenic
PRKN	c.1A>G	p.?		1	Likely Pathogenic
PRKN	c.235G>T	p.Glu79*		1	Pathogenic
PRKN	c.73C>T	p.Gln25*		1	Likely Pathogenic
PRKN	Deletion of Exon 1			1	Pathogenic
PRKN	Deletion of Exons 1-2			1	Pathogenic
PRKN	Deletion of Exons 3-5			1	Pathogenic
PRKN	Deletion of Exon 4-6			1	Pathogenic
PRKN	Deletion of Exon 7			1	Pathogenic
PRKN	Deletion of Exons 8-9			1	Pathogenic
PRKN	Duplication of Exons 1-2			1	Likely Pathogenic
PRKN	Duplication of Exons 1-3			1	Likely Pathogenic
PRKN	Duplication of Exons 1-5			1	Likely Pathogenic
PRKN	Duplication of Exons 2- 4			1	Likely Pathogenic
PRKN	Duplication of Exons 2-3			1	Likely Pathogenic
PRKN	Duplication of Exons 3-6			1	Likely Pathogenic
PRKN	Duplication of Exon 4			1	Pathogenic
PRKN	Duplication of Exon 5-8			1	Likely Pathogenic
PRKN	Duplication of Exons 7-9			1	Likely Pathogenic
SNCA	Whole Gene Duplication			9	Pathogenic
SNCA	c.157G>A	p.Ala53Thr	A53T	1	Pathogenic
PINK1	c.1040T>C	p.Leu347Pro	L347P	6	Pathogenic
PINK1	c.1329del	p.Tyr444Metfs*39		2	Pathogenic
PINK1	c.1366C>T	p.Gln456*		1	Pathogenic
PINK1	c.273del	p.Cys92Alafs*15		1	Pathogenic
PINK1	c.979C>T	p.Gln327*		1	Likely Pathogenic
PINK1	Deletion of Exons 3-8			1	Likely Pathogenic
PARK7	c.471_473del	p.Pro158del	P158del	5	Likely Pathogenic
PARK7	c.105dup	p.Ala36Cysfs*12		1	Pathogenic
PARK7	c.460A>G	p.Thr154Ala		1	Likely Pathogenic
VPS35	c.1858G>A	p.Asp620Asn	D620N	6	Pathogenic

Population frequencies for specific gene variants may vary and can be found through variant databases:
The Genome Aggregation Database (gnomAD)
Trans-Omics for Precision Medicine (TOPMed)
Exome Aggregation Consortium (ExAC)
1000 Genomes Project
The Genome Aggregation Database (gnomAD)
Trans-Omics for Precision Medicine (TOPMed)
NHLBI Exome Sequencing Project (ESP) Exome Variant Server

References

1. Cook L, Verbrugge J, Schwantes-An TH, et al. Providing genetic testing and genetic counseling for Parkinson's disease to the community [published online ahead of print, 2023 Jun 8]. *Genet Med*. 2023;25(10):100907.
2. Richards S, Aziz N, Bale S, et al. Standards and guidelines for the interpretation of sequence variants: a joint consensus recommendation of the American College of Medical Genetics and Genomics and the Association for Molecular Pathology. Genet Med. 2015;17(5):405-424.
