# Supplementary material for: Parkinson’s disease variant detection and disclosure: PD GENEration, a North American study
Source: Brain. 2024 Jul 30;147(8):2668–79. doi: 10.1093/brain/awae142 (PMC11292896; doi:10.1093/brain/awae142)
Supplement: awae142_Supplementary_Data [file awae142_supplementary_data.zip › brain-2023-02602-File010.pdf]

# Parkinson's Foundation and Parkinson Study Group PD GENEration investigators

Collaborators:

Steering Committee:

Roy N. Alcalay, MD, MS[1,4]; Tatiana Foroud, PhD[2]; Anne Hall, JD[3]; Karen Marder, MD, MPH[4]; Ignacio Mata, PhD[5]; Niccolò E. Mencacci, MD[6], PhD; Martha Nance, MD[7]; Michael Schwarzschild, MD, PhD[8]; Tanya Simuni, MD[6]; Anne-Marie Wills, MD[8]

Study Cores:

*Leadership Core:* James C. Beck, PhD[3]; Anna Naito, PhD[3]; Kamalini Ghosh Galvelis, MS[3]; Rebeca De Leon[3]; Amasi Kumeh[3]; Zachary Meyer, MPH[3]; Shilpa Rao, PhD[3]; Anny Coral-Zambrano[3]; Min-Jae Kim[3]; Camilla Ruiz[3]

*Clinical Coordination Center:* Yun Lu, PhD[9]; Elisa Ahmanson, MS[9]; Andra Matthews[9]; Sarah Lawrence, MS[9]

*Data Management Core:* Yun Lu, PhD[9]; Susan Li, MD, PhD[9]; Uma Ragunathan, MS[9]

*Past Clinical Coordination Center:* Cynthia Casaceli, MBA[10]; Deborah Baker[10]; Betty Lyda[10] ; Ken Eaton[10]; Olga Pikul[10]; Ett Muneath[10]; Karen Hodgeman[10]

*Genetics Core and Biorepository:* Yan Meng, PhD[11], Samuel Strom, PhD[11, 66]; Rachel Blake[11]

*Statistics Core*: Tae-Hwi Linus Schwantes-An, PhD[2]

*Genetic Counseling Core*: Tatiana Foroud, PhD[2]; Laura Heathers[2]; Michelle Totten[2]; Lola Cook, MS, CGC[2]; Jennifer Verbrugge, MS, CGC[2]; Jeanine Schulze, MS, CGC[2]; Priscila D. Hodges, MS, CGC[2]; Amanda Miller, MS, CGC[2], Malia Rumbaugh, MS, CGC[2]

Nationally Recruiting Site Investigators:

Niccolò E. Mencacci, MD, PhD[6]; Tanya Simuni, MD[6]; Linn Katus, DO[4]; Joseph Quinn, MD[12]; Tatiana Foroud, PhD[2]

Locally Recruiting Site Investigators:

Rajesh Pahwa, MD[13]; Kelly E Lyons, PhD[13]; Martha Nance, MD[7]; Rossy Cruz Vicioso, MD[14]; Janfreisy Carbonell, MD[14]; William De Jesus, MD[14]; Nora Vanegas Arroyave, MD[15]; Josh Shulman, MD, PhD[15]; Holly Shill, MD[16]; Anne-Marie Wills, MD[8]; Irene Litvan, MD[17];

Thomas Tropea, DO[18]; David Simon, MD[19]; Hubert H. Fernandez, MD[5]; Hemant Pandey, MD[20]; Okeanis Eleni Vaou, MD[21]; Anna Hohler, MD[21]; Carlos Singer, MD[22]; Ihtsham ul Haq, MD[22]; Henry Moore, MD[22]; Stuart Isaacson, MD[23]; Jason Aldred, MD[24]; Ariane Park, MD[25]; Steven Gunzler, MD[26]; Jill Ostrem, MD[27]; Carlie Tanner, MD, PhD[27]; Deborah Hall, MD[28]; Susan Bressman, MD[29]; Rachel Saunders-Pullman, MD[29]; Matthew Barrett, MD[30]; Chantale Branson, MD[31]; Karen Blindauer, MD[32]; Tsao-Wei Liang, MD[33]; Gonzalo Revuelta, MD[34]; Elizabeth Zauber, MD[2]; Julie Schwartzbard, MD[35]; Tritia Yamasaki, MD[36]; Rohit Dhall, MD[37]; Danielle Englert, MD[38]; Giulietta Riboldi, MD[39]; Annie Killoran, MD[40]

Referring Site Investigators:

Kathleen McKee, MD[41]; Nina Browner, MD[42]; Connie Marras, MD[43]; Peter Lin, MD[44]; Irene Richard, MD[45]; Amy Hellman, MD[46]; Gian Pal, MD[47]; Kelly Mills, MD[48]; John Morgan, MD[49]; Mustafa Siddiqui, MD[50]; Jeanne Feuerstein, MD[51]; Jeffrey Cooney, MD[52]; Joseph Savitt, MD[53]; Tarannum Khan, MD[54]; Houman Homayounh, MD[55]; Stephen Lee, MD[56]; Harini Sarva, MD[57]; David Hinkle, MD[58]; Luis Forastieri, MD[59]; Frances Velez, MD[60]; Angel Vinuela, MD[61]; Thomas Beach, PhD[62]; Charles Adler, MD, PhD[63]

Coordinators:

Karen Williams[6]; Max Galarce[6]; Lucas George[6]; Rachel Lewandowski[6]; Evalyn Mackenzie[4]; Amanda Chan[4]; Alia Neibaur[2]; Kellie Keith[12]; Emily Leonard[12]; Gita Golonzka[12]; April Langhammer[13]; Sherry Neisen[7]; Hiba Saade[15]; Jamie Fong[15]; Ruby Rendon[16]; MaryBeth Serrano[5]; Mansi Sharma[5]; Arjun Laud[5]; Isabella Montanaro[5]; Tyler Tribble[5]; Lisa Damron[17]; Neda Almassi[18]; Whitney Hartstone[18]; Aine Russell[19]; Hannah Babcock[19]; Jerrod Cook[5]; Giulia Andrews[20]; Sean Guardado[20]; Kaitlyn Ramsey[21]; Savannah Reis[21]; Claudia Cano[22]; Claire Hennum[23]; Amanda Kiefer[24]; Katherine Ambrogi[25]; Victoria Klee[25]; Victoria Miller[25]; Aaron Daley[27]; Marc Rosenbaum[28]; Deborah Raymond[29]; Maya Rawal[29]; Virginia Norris[30]; Tazrin Rahman[30]; Sherline Sauveur[31]; LaShawn Baker[31]; Paula Phabia-Millbrook[31]; Marie Mejaki[32]; Michelle Rochman[33]; Sandra Wilson[34]; Conner Mroz[2]; Lauren Perrey-Moore[2]; Jonathan Castano[35]; Renee Wagner[36]; Michael Nsoesie[36]; Kimberly Gamble[38]; Kelly Astudillo[39]; Heena Olalde[40]; Min-Jae Kim[3]; Geidy Serrano[62]

Study Partners:

Julia Shirvan, MD, PhD[64]; Judith Peterschmitt, MD, PhD[65]; Pablo Sardi, PhD[65]; Cornelis Blauwendraat, PhD[67]; Christine Klein, MD[68]; Parkinson Study Group

Collaborator Affiliations:

[1] Tel Aviv Sourasky Medical Center, Tel Aviv, Israel

[2] Indiana University School of Medicine, Indianapolis, IN

[3] Parkinson's Foundation, New York, NY

[4] Columbia University, New York, NY

[5] Cleveland Clinic, Cleveland, OH

[6] Northwestern University, Chicago, IL

[7] Park Nicollet Struthers Parkinson's Center

[8] Massachusetts General Hospital, Boston, MA

[9] Navitas Clinical Research, Rockville, MD

[10] Clinical Trials Coordination Center, University of Rochester, Rochester, NY

[11] Fulgent Genetics, Temple City, CA

[12] Oregon Health and Science University, Portland, OR

[13] The Gary A. Smith PD GENEration Site, University of Kansas Medical Center, Kansas City, KS

[14] CEDIMAT,, CECANOT, Unión Médica, Dominican Republic

[15] Baylor College of Medicine, Houston, TX

[16] Barrow Neurological Institute, Phoenix, AZ

[17] University of California, San Diego, CA

[18] University of Pennsylvania, Philadelphia, PA

[19] Beth Israel Deaconess Medical Center, Boston, MA

[20] MD First Research, Chandler AZ

[21] Steward St. Elizabeth Medical Center, Boston, MA

[22] University of Miami, Miami, FL

[23] Parkinson's Disease and Movement Disorders Center of Boca Raton, Boca Raton, FL

[24] Inland Northwest Research, Spokane, WA

[25] Ohio State University, Columbus, OH

[26] University Hospitals Cleveland Medical Center, Cleveland, OH

[27] University of California, San Francisco, CA

[28] Rush University, Chicago, IL

[29] Mount Sinai Beth Israel Medical Center, New York, NY

[30] Virginia Commonwealth University, Richmond, VA

[31] Morehouse College of Medicine, Atlanta, GA

[32] Medical College of Wisconsin, Milwaukee, WI

[33] Jefferson University, Philadelphia, PA

[34] Medical University of South Carolina, Charleston, SC

[35] Aventura Neurologists, Aventura, FL

[36] University of Kentucky, Lexington, KY

[37] University of Arkansas, Fayetteville, AR

[38] Atrium Health, Charlotte, NC

[39] New York University, New York, NY

[40] University of Iowa, Iowa City, IA

[41] Intermountain Healthcare, Salt Lake City, UT

[42] University of North Carolina, Chapel Hill, NC

[43] Toronto Western Hospital, Toronto, Canada

[44] Valley Parkinson Clinic, Los Gatos, CA

[45] University of Rochester, Rochester, NY

[46] University of Nebraska Medical Center, Omaha, NE

[47] Rutgers University, New Brunswick, NJ

[48] Johns Hopkins University, Baltimore, MD

[49] Augusta University, Augusta, GA

[50] Wake Forest Baptist Health, Winston-Salem, NC

[51] University of Colorado, Denver, CO

[52] Duke University, Durham, NC

[53] University of Maryland, Baltimore, MD

[54] Cleveland Clinic Weston, Miami, FL

[55] University of Pittsburgh, Pittsburgh, PA

[56] Dartmouth Hitchcock Medical Center, Lebanon, NH

[57] Weill Cornell Medicine, New York, NY

[58] Ohio Health, Columbus, OH

[59] Dr. Luis J. Forastieri Neurología, Puerto Rico

[60] Clínica Salus, Puerto Rico

[61] Manatí Medical Center, Puerto Rico

[62] Banner Sun Health Research Institute, Sun City, AZ

[63] Mayo Clinic, Phoenix, AZ

[64] Biogen, Boston, MA

[65] Sanofi Genzyme, Cambridge, MA

[66] Illumina, San Diego, CA

[67] National Institute of Health, Bethesda, MD

[68] Institute of Neurogenetics, University of Lübeck, Lübeck, Germany
